# Drug screening for Pelizaeus-Merzbacher disease by quantifying the total levels and membrane localization of PLP1

**DOI:** 10.1016/j.ymgmr.2019.100474

**Published:** 2019-05-07

**Authors:** Takeshi Kouga, Shiro Koizume, Shiho Aoki, Eriko Jimbo, Takanori Yamagata, Ken Inoue, Hitoshi Osaka

**Affiliations:** a Department of Pediatrics, Jichi Medical University, Tochigi, Japan; b Kanagawa Cancer Center Research Institute, Yokohama, Japan; c Department of Mental Retardation and Birth Defect Research, National Institute of Neuroscience, National Center of Neurology and Psychiatry, Kodaira, Japan

**Keywords:** Drug screening, ER-associated degradation, Gene expression, Membrane protein, Oligodendrocyte, PLP1, PMD, Pelizaeus-Merzbacher disease, PLP1, Proteolipid protein 1, ER, Endoplasmic reticulum, *msd*, Myelin synthesis deficient, CNS, Central nervous systems, UPR, Unfolded protein response, EGFP, Enhanced green fluorescent protein, XBP1, X-box binding protein 1, IPA, Ingenuity pathways analysis, IRE1 α, Inositol requiring enzyme 1 α

## Abstract

**Background:**

Pelizaeus-Merzbacher disease (PMD) is caused by point mutations or copy number changes in the proteolipid protein 1 gene (*PLP1*). *PLP1* is exclusively localized in the myelin sheath of oligodendrocytes. Amino acid-substituted PLP1 protein is unable to fold properly and is subsequently degraded and/or restrictedly translated, resulting in a decrease in the PLP1 protein level and a failure to localize to the membrane. Furthermore, misfolded proteins increase the burden on the intracellular quality control system and trafficking, finally resulting in cell apoptosis. The objective of this study was to identify therapeutic chemicals for PMD by quantifying the total levels and membrane localization of PLP1.

**Method:**

We established a cell line stably expressing PLP1^A243V^ fused with green fluorescent protein in oligodendrocyte-derived MO3.13 cells. We screened a chemical library composed of drugs approved for central nervous system disorders that increased both the total intensity of PLP1^A243V^ in the whole cell and the cell membrane localization. We analyzed the change in the endoplasmic reticulum (ER) stress and the gene expression of candidate chemicals using a micro-array analysis. Finally, we tested the in vivo effectiveness using myelin synthesis deficient (*msd*) mice with *Plp*^*A243V*^.

**Results and conclusion:**

Piracetam significantly increased the PLP1^A243V^ intensity and membrane localization and decreased the ER stress. It was also shown to reverse the gene expression changes induced by PLP1^A243V^ in a micro-array analysis. However, in vivo treatment of piracetam did not improve the survival of *msd* mice (Plp1^A243V^).

## 1 Introduction

Proteolipid protein 1 (PLP1: OMIM*300401), composed of 276 amino acid residues, is the most abundant myelin protein in the central nervous systems (CNS) [1,2]. *PLP1*, together with its splice variant *DM20*, is abundantly expressed in maturating oligodendrocytes and slightly expressed in the Schwann cells in the peripheral nerves [3]. PLP1 is synthesized and transported through the endoplasmic reticulum (ER)/Golgi complex where it is correctly folded, processed, and transported to the myelin membrane [4,5]. PLP1 regulates myelin lamellar spacing/compaction and maintains axonal integrity via oligodendrocyte-axonal interactions [6,7].

Pelizaeus-Merzbacher disease (PMD) is an X-linked disease characterized by developmental defects in the myelin sheath formation, mainly in the CNS [5,8]. Overexpression (such as duplication or triplication), deletions/null mutations, or missense mutations of *PLP1*, causes PMD [9]. The overexpression of PLP1 accounts for approximately half of PMD cases, and it is hypothesized that increased levels of PLP1 perturb the assembly of membrane rafts, resulting in the accumulation of PLP1 with cholesterol and lipids in the late endosomal/lysosomal compartments [10,11]. This is reported to cause demyelination and extensive neuronal loss [10]. With loss-of-function mutations (~10%), neurologic dysfunction with diffuse Wallerian degeneration occurs without significant hypomyelination of the CNS axons [12]. The variable severity of PMD in cases involving missense mutations (~30%) is caused by alteration of the *PLP1* structure. When *PLP1* is mutated, the protein is unable to fold properly. Such aberrant proteins cannot travel to the Golgi complex and are consequently trapped in the ER, eliciting the unfolded protein response (UPR) that is related to the pathobiology [13]. Mislocalization of PLP1 also causes a loss of function of the protein, hypomyelination, and a subsequent neurological deficit. In addition, the changes in intracellular localization due to *PLP1* mutations, which consequently cause the premature oligomerization of PLP in the ER, are tightly related to the pathogenesis of PMD [14]. To identify the common pathogenic process underlying PMD, human induced pluripotent stem (iPS) cells and iPS cell-derived oligodendrocytes from PMD patients were generated by Tesar et al. [15]. Recently, iPS cell-derived oligodendrocytes from PMD patients carrying two different missense mutations also showed perinuclear mislocalization of PLP1, in contrast to myelin basic protein, which is a component of myelin that is expressed broadly in cells [16].

In the present study, we explored chemicals that help mutant PLP1 escape ER/lysosomal degradation and reach the cell membrane. These chemicals are also expected to decrease the ER stress. The objective of this study was to screen chemicals for the treatment of PMD based on the expression and intracellular localization of mutant PLP1.

## 2 Materials and methods

### 2.1 Choice of PLP1 mutants and construction of plasmids

The correlation between the position of the amino acid substitutions in *PLP1* and disease severity has been studied [17]. In a previous study, we reported the intracellular distribution of two *PLP1* mutants: PLP1^A243V^ (p.(Ala243Val), severe type) and PLP1^W163L^ (p.(Trp163Leu), mild type) [18]. In addition to these mutants, we selected PLP1^I187T^ (p. (Ile187Thr), mild type, *rumpshaker* in mice and in humans [19]) and PLP1^W163R^ (p.(Trip163Arg), severe type, reported by Hudson et al. [20]) for an analysis.

Plasmids were constructed as previously described, including a fusion protein of enhanced green fluorescent protein (EGFP), pPLP-EGFP (pPLP1^WT^-EGFP, pPLP1^A243V^-EGFP, pPLP1^W163L^-EGFP), and PLP1 with FLAG peptide tag (pPLP1^WT^-FLAG, pPLP1^A243V^-FLAG) [18]. In short, full-length cDNA of human *PLP1* was amplified by a reverse transcription polymerase chain reaction (RT-PCR) and cloned into the pEGFP-N1 vector (Clontech Laboratories, Inc., Santa Clara, CA, USA) at the *Eco*RI/BamHI site to produce pPLP1-EGFP. pPLP1-FLAG was derived from pPLP1-EGFP plasmids by inserting a FLAG tag nucleotide sequence between the PLP1 and EGFP. The plasmids of pPLP1^W163R^-EGFP and pPLP1^I187T^-EGFP that were used in this study were also prepared by the same procedure [18]. The primers used for strand synthesis were 5′-CCTGACCGTTGTGCGGCTCCTGGTG-3′and 5′-CACCAGGAGCCGCACAACGGTCAGG-3′ (for W163R conversion), and 5′-CCTGCCAGTCTACTGCCTTCCCCAGC-3′ and 5′-GCTGGGGAAGGCAGTAGACTGGCAGG-3′ (for I187T conversion).

### 2.2 Maintenance of MO3.13 cells

The MO3.13 cells derived human oligodendrocyte were maintained in complete growth medium consisting of Dulbecco's minimal essential medium (DMEM: high glucose) (Invitrogen, Carlsbad, CA, USA) and 10% fetal bovine serum (ThermoFisher Scientific, Waltham, MA, USA). The cells were incubated in a humidified incubator with an atmosphere of 5% CO_2_, set at 37 °C and passaged no >10 times, grown to confluence in 60-mm culture dishes.

### 2.3 MO3.13 cells stably expressing PLP1-EGFP

To measure the expression and intracellular localization of wild and mutant types of PLP1-EGFP, we transfected PLP1^WT^ and mutant PLP1 plasmids (PLP1^A243V^, PLP1^W163R^, PLP1^I187T^, and PLP1^W163L^) fused with EGFP to MO3.13 cells using the Lipofectamine® 3000 Transfection Kit (Thermo Fisher Scientific). Next, we created a stable cell line using G418 (Geneticin®), an aminoglycoside related to Gentamicin for each PLP1 plasmid. For colony selection, we seeded MO3.13 cells derived from a colony onto 8-well chamber plates at 2 × 10^5^ cells per well and confirmed EGFP fluorescence by confocal microscopy (LUOVIEW FV1000; Olympus, Tokyo, Japan).

### 2.4 The analysis of the PLP1^A243V^-EGFP fluorescence intensity in the whole cell using a chemical library

We adopted a CNS library (PerkinElmer, Inc., Waltham, MA, USA) for drug screening. This library contained 275 structurally diverse FDA-approved drugs that affect the CNS (Prestwick Chemical Library®) [21]. Because misfolded proteins are subject to ER/Golgi degradation and decrease the total amount of PLP1 [18], we examined the intensity of PLP1^A243V^-EGFP in the whole cell before and after adding this library. The fluorescence intensity of PLP1^A243V^-EGFP was analyzed using an In Cell Analyzer 1000® (GE Healthcare UK Ltd., Amersham, UK), which is a modular, cellular and subcellular imaging system for automated imaging and analyses. First, we seeded MO3.13 cells stably expressing PLP1^A243V^ onto 96-well plates at 1 × 10^4^ cells per well. Twenty-four hours later, the whole-cell EGFP fluorescence intensity was analyzed using an In Cell Analyzer 1000® with a protocol that analyzes the fluorescence intensity in the whole cell and the baseline figures for each well. In the analysis, 5 nuclei (Hoechst 33342) and EGFP image fields (each containing about 20 cells) were automatically acquired per well (Nikon 20× Planfluor objective) and subjected to an automated image analysis using the In Cell Analyzer 1000 Workstation 3.4 software program (GE Healthcare UK Ltd., Amersham, UK) [22]. Subsequently, 275 different chemicals were added at a final concentration of 10 μM each. Each chemical was dissolved in Phosphate buffered salts (PBS), Dimethyl sulfoxide (DMSO), ethanol or chloroform depending on the solubility. The next day, the fluorescence intensity of EGFP was analyzed by the In Cell Analyzer 1000® with the same protocol and compared to the fluorescence intensity before chemicals were added. Then the after/before intensity ratio was calculated. The ratio of fluorescence intensity with each chemical was corrected by the ratio for control cells (analyzed at the same two points, with the same amount of solvent added for each chemical).

### 2.5 The analysis of PLP1^A243V^-EGFP in the cell membrane using confocal microscopy images

To analyze the intensity of PLP1-EGFP in the cell membrane, which reflects the appropriate intracellular localization of the expressed protein, we examined the fluorescence intensity in the cell membrane of PLP1^A243V^-EGFP on images obtained using a confocal microscope. An In Cell Analyzer 1000® was used for the 9 chemicals that were associated with a >1.5-fold in the whole cell EGFP intensity. We randomly took five images from fixed (with 4% paraformaldehyde for 60 min at room temperature) MO3.13 cells that were observed to stably express PLP1^A242V^-EGFP under a confocal microscope (FLUOVIEW FV1000; Olympus) 24 h after adding chemicals at a final concentration of 10 μM. Each picture contained an average of 27.2 cells. We then analyzed the fluorescence intensity of the cell membrane using an In Cell Analyzer 1000® with a protocol that defines the cell membrane fraction as the outer region of each cell (Supplementary Fig. A). In the protocol, configuration of the membrane thickness was set after the erosion process from the cell surface based on the specified cytoplasm area [23]. For candidate chemicals that were associated with a significant increase in fluorescence intensity at the cell membrane, we also compared the intensity of EGFP at the cell membrane before and after the addition of the candidate chemicals for other mutant proteins fused with EGFP from cells stably expressing PLP1^W163R^, PLP1^I187T^, and PLP1^W163L^.

### 2.6 The analysis of the ER stress reaction

We examined whether or not the correction of localization reduced the ER stress that had been at least partly implicated in the pathophysiology of PMD [15]. We incubated MO3.13 cells stably expressing PLP1^A243V^-EGFP in 96-well plates at 2 × 10^4^ cells per well and separately added the candidate chemicals piracetam and benserazide at a final concentration of 10 μM. We also incubated MO3.13 cells that stably expressed PLP1^WT^-EGFP under the same conditions. After 24 h, we transfected these cells with an ER stress detector plasmid (ERAI ER Stress Detector®; Cosmo Bio Co., Ltd. Tokyo, Japan) by the lipofectamine method. This detector contained an *XBP1* gene fused with luciferase. Twenty-four hours later, we exchanged the medium for one containing tunicamycin (5 μg/ml), which inhibits glycoprotein synthesis and induces ER stress via an inositol-requiring enzyme 1α (IRE1α) protein sensor. IRE1α is an ER-located transmembrane protein that evokes *XBP1* gene splicing depending on the ER stress imposition [24]. As a result of *XBP1* gene splicing, the fused luciferase is translated and detected. Six hours after the addition of tunicamycin, we measured the luciferase intensity using a ONE-Glo™ Luciferase Assay System® (Promega, Madison, WI, USA).

### 2.7 PLP1^A243V^-FLAG and ER staining

Because adding EGFP proteins might alter the PLP localization [25], we tested the efficacy of piracetam using PLP1^A243V^-FLAG. As reported previously [26], this mutant protein accumulates in the ER. Thus, we examined the co-localization of PLP1 and proteins in the ER using the anti-KDEL antibody [27]. First, we incubated MO3.13 cells stably expressing PLP1^A243V^-FLAG in 8-well chamber plates at 2 × 10^5^ cells per well. We then added one of the candidate chemicals extracted above, piracetam, at 10 μM. After incubation at 37 °C for 24 h, cells were fixed in 4% paraformaldehyde for 60 min at room temperature. Following fixation, the cells were washed 3 times with PBS and rinsed with blocking buffer (5% skimmed milk/PBS containing 0.1% triton X-100) for 60 min at room temperature and then incubated with a primary antibody (containing blocking buffer) overnight at 4 °C. The following primary antibodies were used: mouse monoclonal anti-FLAG (OriGene Technologies Inc., Rockville, MD, USA) at 1:200 and rabbit polyclonal anti-KDEL (Medical & Biological Laboratories Co., Ltd., Nagoya, Japan) at 1:1000. After 3 PBS washes with 0.1% triton X-100 (PBS-T), immune-complexes were detected using the following secondary antibodies (60 min at room temperature): goat anti-mouse IgG Alexa Fluor® 488 and goat anti-rabbit IgG Alexa Fluor® 568 (Thermo Fisher Scientific, Waltham, MA, USA) at 1:250. Hoechst 33342 was then used to visualize nuclei. We performed the same procedure for PLP1^A242V^-FLAG without piracetam and PLP1^WT^-FLAG. Fluorescence staining was observed using a confocal microscope (FLUOVIEW FV1000; Olympus) at x600.

### 2.8 A microarray analysis of the changes in the gene expression induced by candidate chemicals

We investigated the changes in the overall gene expression in MO3.13 cells induced by PLP1^A243V^ and piracetam by RNA microarray analyses. Using an RNeasy® Mini kit (QIAGEN N.V., Venlo, Netherlands), we extracted 3 types of RNA from MO3.13 cells: stably expressing PLP1^A243V^ 24 h after adding piracetam at 10 μM, PLP1^A243V^ without piracetam, and PLP1^WT^. To assess the gene expression, we performed a microarray analysis that included labeling, hybridization, scanning, and data processing (Agilent Technologies Inc., Santa Clara, CA, USA) with the SurePrint G3 Human GE V3 8x60k platform (Agilent Technologies Inc.). The microarray data has been deposited in GEO database (GEO Accession Number: GSE 124034). From the gene probes on the platform (58,201 probes), we extracted the genes whose expression increased more than two-fold (increased group) or decreased to less than half (decreased group) after normalization of the microarray and extracted “Detected probes (possessing high reliability)” using the GeneSpring GX 14.5 software program (Agilent Technologies Inc.). We then imported these genes into the IPA software program (version 44,691,306; QIAGEN N.V., Venlo, Netherlands) and exported the canonical pathways to which the extracted genes significantly contributed.

### 2.9 Administration of piracetam to msd mice

We assessed whether or not piracetam could improve the phenotype of a naturally occurring Plp1^A243V^ mouse model, myelin synthesis deficiency (*msd*) mouse”. We treated wild-type and *msd* mice with piracetam and vehicle to examine the effect of piracetam on body weight and survival. All *msd* mice were male. Piracetam (CAS No. 7491-74-9) was purchased from Sigma-Aldrich Co., LLC (St. Louis, MO, USA). For the treated group (*N* = 14), 200 mg/kg of piracetam dissolved in PBS (final concentration of piracetam: 60 mg/ml) was administered by intraperitoneal injection for 5 consecutive days per week from postnatal day 3. For the control group (*N* = 24), the same amount of PBS was administered in the same pattern. The dosage of piracetam was determined by referencing previous reports [[28], [29], [30]]. All of the animal handling and treatment protocols were reviewed and approved by the Animal Care and Use Committee of National Institute of Neuroscience, National Center of Neurology and Psychiatry (approval number, 2017010).

### 2.10 Statistical analyses

Data are expressed as the mean ± standard error of the mean. Student's *t*-test was used for comparisons between two groups, and the Gehan-Breslow-Wilcoxon test was used for the survival analysis. The significance level for statistical comparison was *P* < 0.05.

## 3 Results

### 3.1 The stable expression of PLP1-EGFP in an MO3.13 cell line

**Fig. 1:**
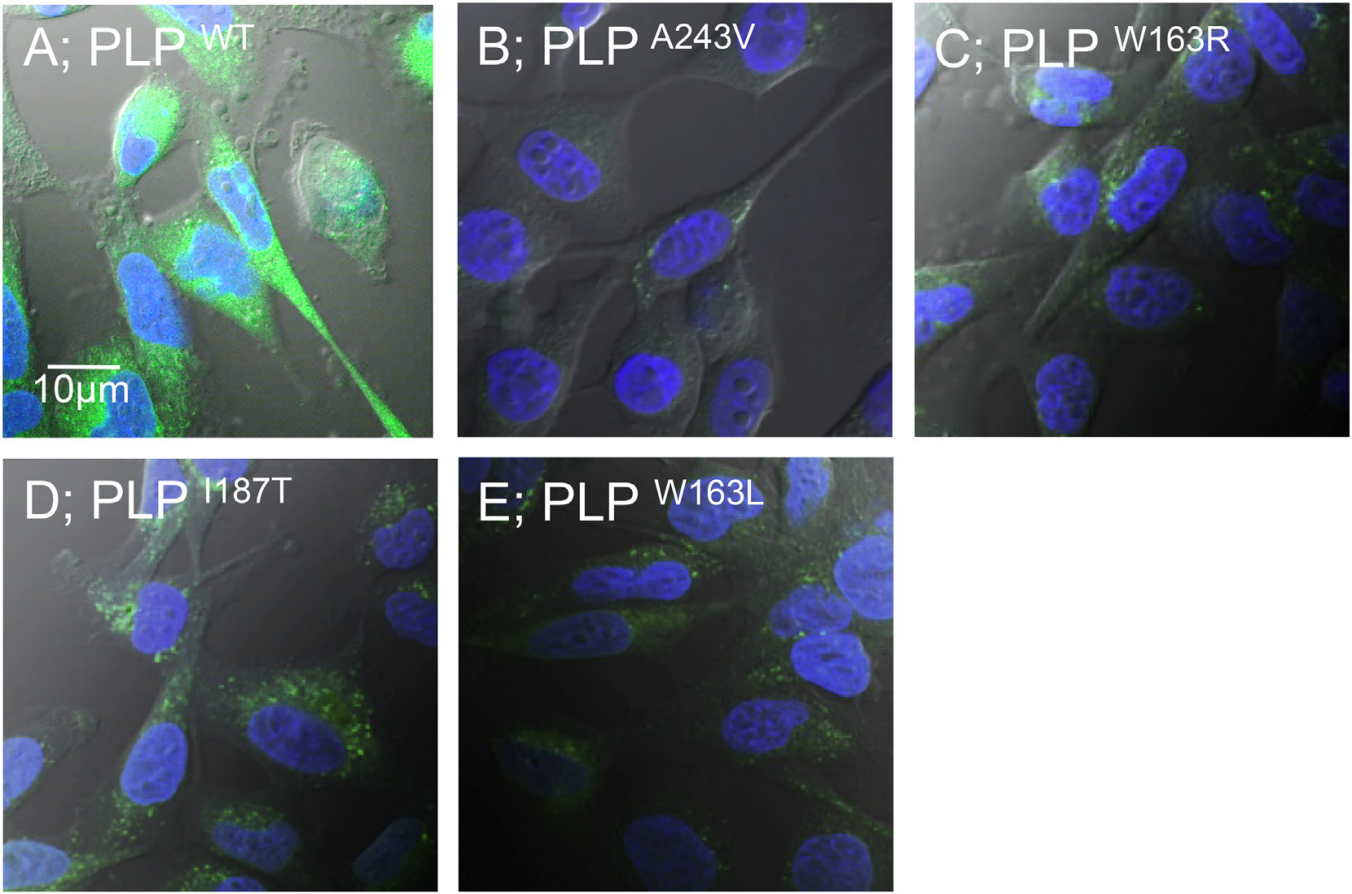
The results of measuring the expression and intracellular localization of wild and mutant types of PLP1-EGFP, images of MO3.13 cells stably expressing PLP1 wild-type (Panel A) and four types of PLP1 mutants fused with EGFP: PLP1^A243V^ (B), PLP1^W163R^ (C), PLP1^I187T^ (D), and PLP1^W163L^ (E) are shown. Fused EGFP protein is shown in green, and Hoechst-stained nuclei are shown in blue. Each image was taken by a confocal microscope (magnitude, ×600). PLP1^WT^ was distributed strongly throughout the cells (A). Severe-type mutants (PLP1^A243V^ and PLP1^W163R^) showed a limited expression, including in the perinuclear region (B, C). Mild-type mutants (PLP1^I187T^ and PLP1^W163L^) showed an increased expression compared to severe-type mutants but less restricted localization than wild-type specimens (D, E). (For interpretation of the references to colour in this figure legend, the reader is referred to the web version of this article.)

To measure the expression and intracellular localization of wild and mutant types of PLP1-EGFP, MO3.13 cells stably expressing PLP1 wild-type and four types of PLP1 mutants fused with EGFP: PLP1^A243V^, PLP1^W163R^, PLP1^I187T^, and PLP1^W163L^ were established (Fig. 1). PLP1^WT^ was broadly distributed in the cytoplasm and cell membrane, as we have already shown in COS-7 cells derived from monkey kidney tissue (Fig. 1A; [13]). The mutants PLP1^A243V^ and PLP1^W163R^ showed the weak expression and accumulation of PLP1 in the perinuclear region in most cells (Fig. 1B, C). The mild-type mutants PLP1^I187T^ and PLP1^W163L^ showed stronger expression levels and a less-restricted localization pattern in comparison to the severe mutants (Fig. 1D, E). Among these PLP1 mutations, PLP1^A243V^ has been reported in cases of severe PMD. In addition, the model mouse, *msd* mouse (Plp1^A243V^) exists in this mutation [31]. We therefore selected cells stably expressing the PLP1^A243V^ mutation for drug screening.

### 3.2 In-cell analyses of the whole-cell PLP1-EGFP fluorescence intensity with chemicals

**Fig. 2:**
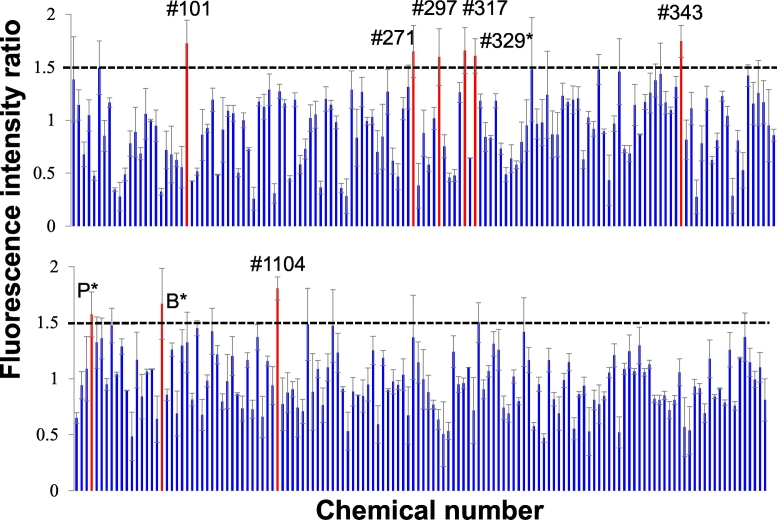
The results of analyses of the change in PLP1-EGFP intensity in the whole cell with chemicals, ratios of the whole-cell EGFP fluorescence intensity in MO3.13 cells stably expressing PLP1^A243V^ 24 h before/after adding 275 chemicals analyzed by an In Cell Analyzer 1000® are shown. Each ratio is the result of averages from an analysis of each of three biological replicates with an In Cell Analyzer® and has been corrected by the ratio of control cells (analyzed at the same two points, with addition of the same amount of solvents for each chemical). Chemicals that showed a > 1.5-fold fluorescence intensity are highlighted in red. Error bar: mean ± standard error (SE). #101, apomorphine; #271, vincamine; #297, benzydamine; #317, guanethidine; #329, tacrine; #343, desipramine; P, piracetam; B, benserazide; #1104, clonixin lysinate. The chemical number denoted by an asterisk means that the fluorescence intensity with the concerned chemical shows a *P*-value <.05 compared with control. For these 9 chemicals, all chemicals were dissolved by DMSO, with the exception of vincamine, which was dissolved by chloroform. (For interpretation of the references to colour in this figure legend, the reader is referred to the web version of this article.)

The ratios of the whole-cell EGFP fluorescence intensity in MO3.13 cells stably expressing PLP1^A243V^ 24 h after to that before adding 275 chemicals were analyzed using an In Cell Analyzer 1000® (Fig. 2). The solvent of each chemical was DMSO, with the exception of vincamine, which was dissolved by chloroform. An analysis of each of 3 biological replicates with the In Cell Analyzer revealed that 9 chemicals showed a >1.5-fold increase in EGFP intensity: #101, apomorphine; #271, vincamine; #297, benzydamine; #317, guanethidine; #329, tacrine; #343, desipramine; P, piracetam; B, benserazide; and #1104, clonixin lysinate.

### 3.3 Cell membrane localization analyses for PLP1-EGFP with chemicals

**Fig. 3:**
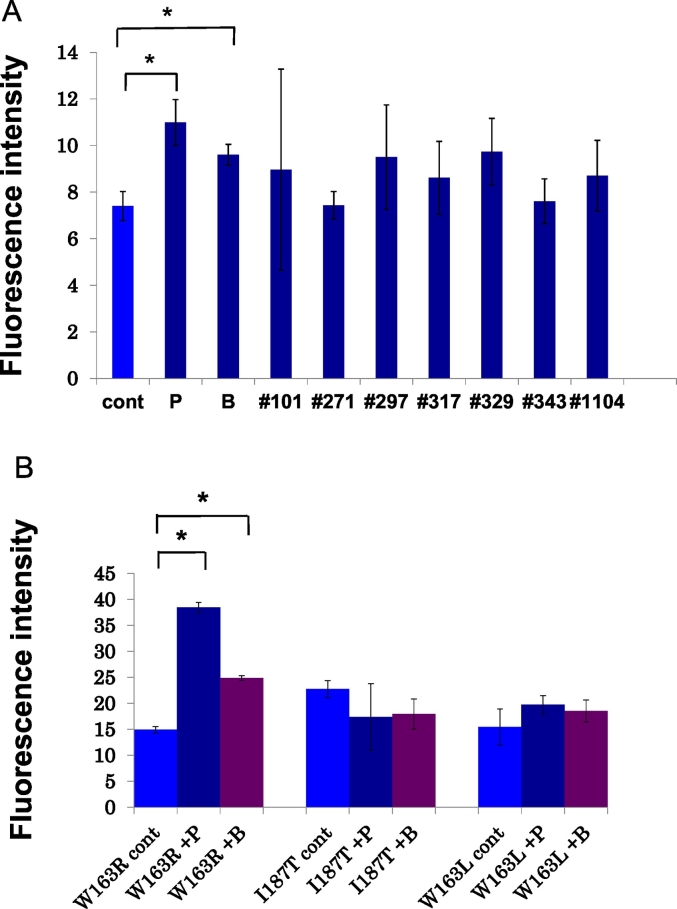
The results of analyses of the cell membrane localization of PLP1-EGFP and the EGFP intensities of PLP1 mutants with chemicals are shown. (A) The EGFP fluorescence intensities of PLP1^A243V^ in the cell membrane after the addition of 9 chemicals extracted in the first step were analyzed with an In Cell Analyzer 1000®. Among these, piracetam (P, *P* = 0.01) and benserazide (B, *P* = 0.009) significantly increased the fluorescence intensity at the cell membrane. The intensity of the other seven chemicals (#101, apomorphine; #271, vincamine; #297, benzydamine; #317, guanethidine; #329, tacrine; #343, desipramine; #1104, clonixin lysinate) was not markedly affected. (B) The EGFP fluorescence intensity of PLP1^W163R^, PLP1^I187T^, and PLP1^W163L^ at the cell membrane after the addition of piracetam and benserazide. Both chemicals significantly increased the fluorescence intensity at the cell membrane in PLP1^W163R^. *P < 0.05. Error bar: mean ± SE.

The results of an analysis of the cell membrane localization of PLP1-EGFP showing the EGFP intensities of PLP1 mutants with chemicals are presented. The EGFP fluorescence intensities of PLP1^A243V^ at the cell membrane after adding the 9 chemicals extracted in the first step were analyzed with an In Cell Analyzer 1000® (Fig. 3A). An analysis of 5 biological replicates for each of 9 chemicals (45 pictures in total) showed that the mean intensity of EGFP in the cell membrane with piracetam and benserazide was significantly higher than in control cells. For other PLP1 mutants, the severe mutant PLP1^W163R^ also significantly increased the intensity of EGFP in the cell membrane (Fig. 3B). However, the mild-type mutants PLP1^I187T^ and PLP1^W163L^ did not show a significant increase in the membrane fraction.

### 3.4 ER stress changes by piracetam and benserazide in MO3.13 cells stably expressing PLP1^A243V^

**Fig. 4:**
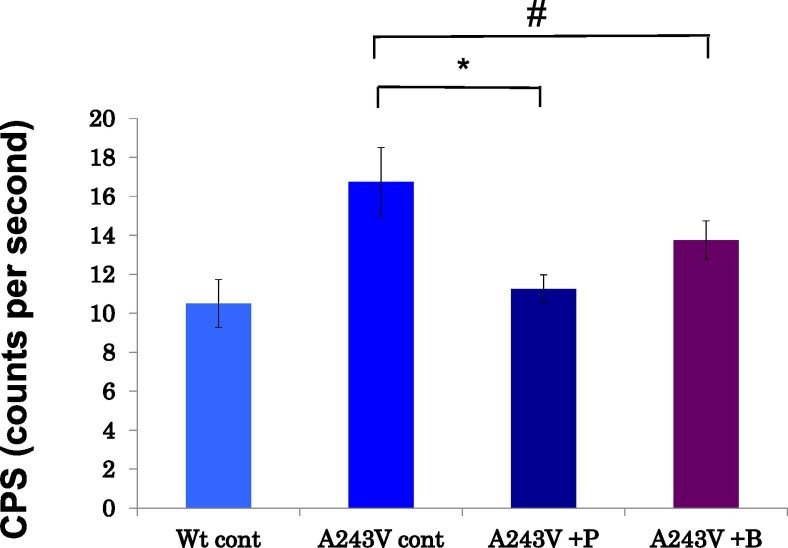
The results of an examination as to whether or not the correction of localization reduced the ER stress and the ER stress activity as determined based on the luciferase assay of PLP1^A243V^ after the addition of piracetam and benserazide and PLP1^WT^ are shown. The CPS is the index of the ER stress activity that reflects the luciferase activity of a spliced *XBP1* RNA. Piracetam significantly decreased the ER stress activity (*P* = 0.04). Although no significant difference was noted, benserazide tended to reduce the ER stress (*P* = 0.07). *P < 0.05. #*P* = 0.07. Error bar: mean ± SE.

We examined whether or not the correction of localization by chemicals reduced the ER stress. After transfecting an ER stress detector that contained the *XBP1* gene fused with luciferase, we measured the induction of luciferase caused by *XBP1* gene splicing (which reflects ER stress) twice on each of three biological replicates using cells stably expressing PLP1^A243V^. Piracetam significantly reduced *XBP1* gene splicing, indicating that this chemical decreased the ER stress. However, the reduction induced by benserazide was not significant (Fig. 4). Thus, we studied piracetam in the subsequent analyses.

### 3.5 Piracetam improved the intracellular localization of PLP1^A243V^

**Fig. 5:**
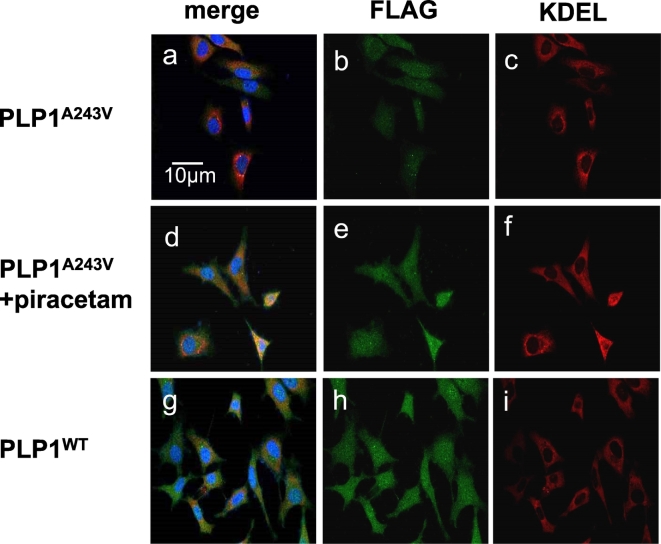
The results of an examination of the efficacy of piracetam with co-localization of PLP1 and proteins in the ER, images of MO3.13 cells stably expressing PLP1^A243V^-FLAG and PLP1^WT^-FLAG with anti-KDEL antibody (×600) are shown. PLP1^A243V^-FLAG and PLP1^WT^-FLAG are shown in green (b, e, h), and KDEL, which reflects ER localization (c, f, i), is shown in red. PLP1^A243V^ proteins are weakly expressed mainly in the ER (a-c). After the addition of piracetam, PLP1^A243V^ was localized throughout the intracellular region (d-f). Nuclei stained with Hoechst are shown in blue. a-c: PLP1^A243V^, d-f: PLP1^A243V^ with piracetam, and g-i: PLP1^WT^. (For interpretation of the references to colour in this figure legend, the reader is referred to the web version of this article.)

We examined the efficacy of piracetam for the co-localization of PLP1 and proteins in the ER using the anti-KDEL antibody. Images of MO3.13 cells stably expressing PLP1^A243V^-FLAG, PLP1^A243V^-FLAG after the addition of piracetam and PLP1^WT^-FLAG with anti-KDEL antibodies are shown in Fig. 5 (×600). Analyses of one biological replicate showed that PLP1^A243V^-FLAG was weakly expressed in comparison to the wild type and was mainly localized in the ER (Fig. 5; a-c vs. g-i). After treatment with piracetam, PLP1^A243V^-FLAG proteins were localized throughout the cell, indicating that the localization of PLP1^A243V^-FLAG was similar to that of the wild type (Fig. 5; d-f).

### 3.6 Piracetam changed the gene expression in MO3.13 cells with PLP1^A243V^

We investigated the gene expression changes in MO3.13 cells induced by PLP1^A243V^ and piracetam. Among the 58,201 probes on the microarray platform SurePrint G3 Human GE V3 8x60k (Agilent Technologies Inc.), the numbers of valid probes for each RNA (PLP1^A243V^, PLP1^A243V^ with piracetam, and PLP1^WT^) were 28,715, 28,786, and 28,692 probes, respectively. For these, we compared the log2 ratio used as a measure of the gene expression between PLP1^WT^ and PLP1^A243V^. We then compared the expression profile in MO3.13 cells that stably expressed PLP1^A243V^ with/without piracetam (Table 1; e.g., a log2 ratio of 4 means 16 times higher than control, while a log2 ratio of −4 means 1/16 of the control). By adding piracetam, the gene expression was increased >2-fold or decreased to less than half in 3890 genes. Among these, piracetam altered the expression of 1549 genes (39%) in MO3.13 cells stably expressing PLP1^A243V^ in the opposite direction of the changes from PLP1^WT^ to PLP1^A243V^ (Table 2). A heat map representing the colour-coded expression levels of the genes in MO3.13 cells that are stably expressed in PLP1^WT^, PLP1^A243V^ and PLP1^A243V^ with piracetam is shown in Supplementary Fig. B. In a canonical pathway analysis through an ingenuity pathways analysis (IPA), we extracted the top five altered pathways (separated into decreased and increased expression groups) to which genes that showed expression levels that were altered by piracetam highly contributed (Table 3).

**Table 1:** The top 20 altered genes in MO3.13 cells stably expressing PLP^A243V^ with and without piracetam.

Decreased genes		Increased genes	
Gene symbol	Log2 ratio	Gene symbol	Log2 ratio
MT1H	−5.51	*SOX1*	4.94
CD69	−5.39	*HIGD2B*	4.84
NAV2-AS5	−5.18	*PSMA8*	4.74
INSL5	−5.15	*CABP2*	4.70
WSCD2	−5.08	*SPARCL1*	4.59
KIF19	−5.00	*TSPAN8*	4.56
TNMD	−4.98	*GRIA3*	4.53
DKFZP434A062	−4.78	*RTP2*	4.53
DNAJB5-AS1	−4.60	*MMP26*	4.45
HOTAIR	−4.60	*PDZK1IP1*	4.43
F13A1	−4.57	*PLA2G12B*	4.39
RNF222	−4.56	*SNORA35*	4.36
OR10J1	−4.51	*DNMBP-AS1*	4.30
GPR45	−4.48	*MIR7515HG*	4.21
SPATA13-AS1	−4.38	*CRP*	4.13
DNASE1	−4.34	*PLA2G10*	4.11
TSRM	−4.32	*HLA-DRB6*	4.10
RBMY3AP	−4.29	*AADACL3*	4.10
SPOPL	−4.29	*MUC19*	4.10
OR5P3	−4.24	*SERPINC1*	4.04

From differentially expressed genes in microarray analysis, the top 20 altered genes in MO3.13 cells stably expressing PLP^A243V^ with piracetam (10 μM) are shown. The left panel shows the decreased genes, and the right panel shows the increased genes compared with those in cells stably expressing PLP1^A243V^ without piracetam. The underlined genes are those whose changes in expression were reversed by switching from a PLP1^WT^ to a PLP1^A243V^ mutation.

**Table 2:** Correspondence table of Log2 ratio for the underlined genes in Table 1 with gene expression intensity in PLP1^A23V^ compared with PLP1^WT^.

	Gene expression change in PLP1^A243V^ with piracetam	Gene expression intensity in PLP^A243V^ compared with PLP1^Wt^
MT1H	−5.51	5.38
CD69	−5.39	5.24
NAV2-AS5	−5.18	4.59
INSL5	−5.15	5.02
WSCD2	−5.08	5.00
KIF19	−5.00	3.18
DKFZP434A062	−4.78	3.52
DNAJB5-AS1	−4.60	4.46
F13A1	−4.57	4.45
RNF222	−4.56	4.45
OR10J1	−4.51	4.75
GPR45	−4.48	3.98
SPATA13-AS1	−4.38	4.26
DNASE1	−4.34	4.80
TSRM	−4.32	4.20
RBMY3AP	−4.29	4.16
SPOPL	−4.29	3.68
OR5P3	−4.24	4.12
SPARCL1	4.59	−2.15
GRIA3	4.53	−1.32
MUC19	4.10	−3.64

Correspondence table of Log2 ration for underlined genes in Table 2 whose expression reversed by piracetam from the change of a PLP1^WT^ to a PLP1^A243V^ mutation. Of 3890 genes that increased >2 times or decreased less than half by poracetam, 1549 genes (39%) expression showed reversed gene expression.

**Table 3:** The top five altered pathways in an ingenuity pathways analysis with piracetam.

Decreased group	Increased group
Asparagine degradation (0.01)	Phospholipases (0.003)
Alanine degradation (0.01)	Polyamine regulation in colon cancer (0.005)
Alanine biosynthesis (0.01)	Synaptic long term depression (0.009)
Coagulation system (0.01)	Macrophage migration inhibitory factor (MIF)-Mediated glucocorticoid regulation (0.01)
Proline degradation (0.01)	Antioxidant action of vitamin C (0.01)

Genes in the decreased and increased groups are those whose expression decreased to less than half or increased more than two-fold, respectively, following piracetam treatment. Figures in parentheses indicate the *P* value from comparing the gene expression with or without piracetam that contributed the concerned pathway.

### 3.7 In vivo *treatment of piracetam did not mitigate the survival of* msd *mice*

**Fig. 6:**
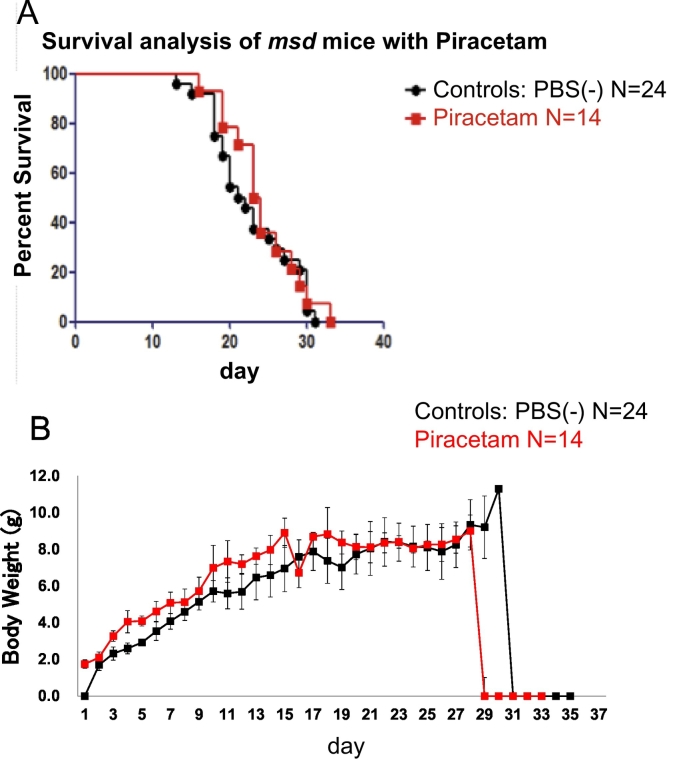
The results of an assessment as to whether or not piracetam improves the phenotype of *msd* mice, the results of a survival analysis (A) and the body weight (B) of piracetam-treated *msd* mice are shown. For the treated group (*N* = 14), 200 mg/kg of piracetam dissolved in PBS (final concentration of piracetam: 60 mg/ml) was administered by intraperitoneal injection for 5 consecutive days per week from postnatal day 3. For the control group (*N* = 24), the same amount of PBS was administered in the same pattern. There were no significant differences between the treated group (N = 14) and the control group (N = 24). The results were statistically analyzed using the Gehan-Breslow-Wilcoxon test (*P* = 0.38).

We assessed whether or not piracetam improved the phenotype of *msd* mice. The results of a survival analysis (Fig. 6A) and body weight (Fig. 6B) in piracetam-treated *msd* mice are shown. There was no significant difference in the survival or body weight of the piracetam-treated group and the control group. The mean survival duration of the piracetam-treated group and control group was 23.5 and 21.5 days, respectively (*P* = 0.38 [Gehan-Breslow-Wilcoxon test]).

## 4 Discussion

At present, there are no effective treatments for PMD [32]. Although gene replacement therapy using a viral vector has been applied for genetic neurological diseases, simple replacement cannot be used to treat PMD caused by gain-of-function mutant *PLP1*. We searched for small molecules that improve the cellular localization of mutant PLP1 in stably transfected MO3.13 cells by visualizing the cellular localization of PLP1 and subsequently identified piracetam. We showed here that piracetam increased the protein expression and membrane localization in two mutations (PLP1^A243V^ and PLP1W^163R^) found in severe cases of PMD. In the cell membrane localization analyses, the mild-type mutants PLP1^I187T^ and PLP1^W163L^ did not show a significant increase in the membrane fraction. One reason for this result may be that these mutants were originally located broadly due to the rapid degradation of the ER or were degraded by another pathway, such as a proteasome- or lysosome-dependent pathway [26]. The localization of these mutant proteins was therefore largely unaffected by piracetam that have been shown to reduce ER stress in this study.

Piracetam is a cyclic derivative of γ-aminobutyric acid. It modulates neurotransmission, including the cholinergic and glutamatergic systems, and has neuroprotective and anticonvulsant properties [33]. Its efficacy has been documented in various neuronal diseases, including cortical myoclonus [34]. At the cell membrane in the aged brain, reduced cell membrane fluidity is caused by an increased ratio of cholesterol to phospholipids due to an increased amount of saturated fatty acids [35]. The chronic treatment of aged rats with piracetam decreased brain cell membrane anisotropy [36]. These results suggest that piracetam acts at the polar head of the phospholipid bilayer and modifies the anisotropy of the cell membrane that results in reduced membrane fusion (i.e., piracetam has rheological properties) [37]. In cells with mutant PLP1, the membrane fluidity at the ER and Golgi complex is expected to be reduced by the accumulation of cholesterol bound to mutant PLP1. This action of piracetam may be related to the changes in the membrane fluidity at the ER and Golgi complex and therapeutic properties in PLP1 mutants.

Regarding the gene expression, piracetam altered the expression of genes in MO3.13 cells stably expressed PLP1^A243V^ toward the opposite direction of the change from PLP1^WT^ to PLP1^A243V^, thereby reversing the changes induced by the PLP1^A243V^ mutation. This suggests that piracetam has compensatory or inhibitory effects against the deleterious changes induced by the PLP1^A243V^ mutation. In a canonical pathway analysis, several pathways reversed by piracetam were found to have possible involvement in ER stress (Table 3). First, IPA showed that piracetam decreased the activity of the proline degradation pathway, which led to an increase in the amount of proline. The genetic depletion of proline biosynthesis reportedly decreased the ER stress tolerance, and proline is considered critical for maintaining the intracellular redox environment under conditions of ER stress [38]. Second, the phospholipase pathway, which was enhanced by piracetam, has also been reported to modulate ER stress through cytosolic phospholipase A2-α [39]. Third, piracetam enhanced the pathway underlying the antioxidant action of vitamin C, which protects mice from cadmium-triggered germ cell apoptosis by inhibiting ER stress related to IRE1α and UPR [40].

However, despite improvements in the PLP1 mutant localization in stably transfected MO3.13 cells, the decrease in splicing of *XBP1* RNA, and the improvement in the gene expression, we were unable to show the significant elongation of the life span of our model mice. In this study, the piracetam dose was determined by referencing previous reports. Administering a higher dose of piracetam might be suitable [33]. In addition, assessments of the brain tissue and behavioral changes in *msd* mice treated with piracetam should be performed in future studies.

In summary, we screened drugs for PMD where the pathogenicity is identified by a decreased amount of protein and localization changes related to evoked ER stress. We successfully identified piracetam, which increased the amount of protein and enhanced the proper localization of PLP1^A243V^. Piracetam also reduced the ER stress for PLP1^A243V^. However, we failed to demonstrate the in vivo effectiveness, and further derivatization is required.

## Conflict of interest statement

The authors declare no conflicts of interest in association with the present study.

## Appendix A Supplementary data

Supplementary material Image 1
