# Supplementary material for: Drug screening for Pelizaeus-Merzbacher disease by quantifying the total levels and membrane localization of PLP1
Source: Mol Genet Metab Rep. 2019 May 7;20:100474. doi: 10.1016/j.ymgmr.2019.100474 (PMC6510973; doi:10.1016/j.ymgmr.2019.100474)
Supplement: Supplementary file 1 — Supplementary material [file mmc1.docx]

**
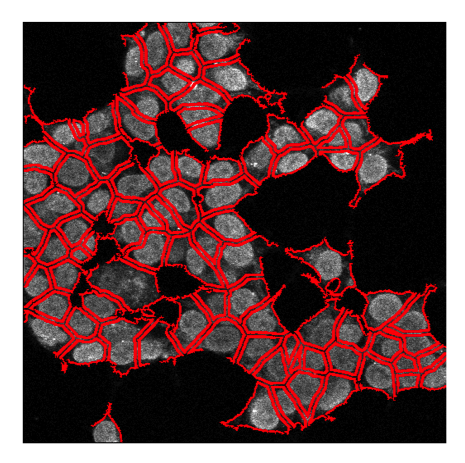
Supplementary Figure A.** A representative image of MO3.13 cells in which the cell membrane region (recognized by an In Cell Analyzer 1000^®^) are expressed in red (x200). The red region is derived from the In Cell Analyzer program, wherein the cell membrane regions are set after the erosion process from the cell surface.


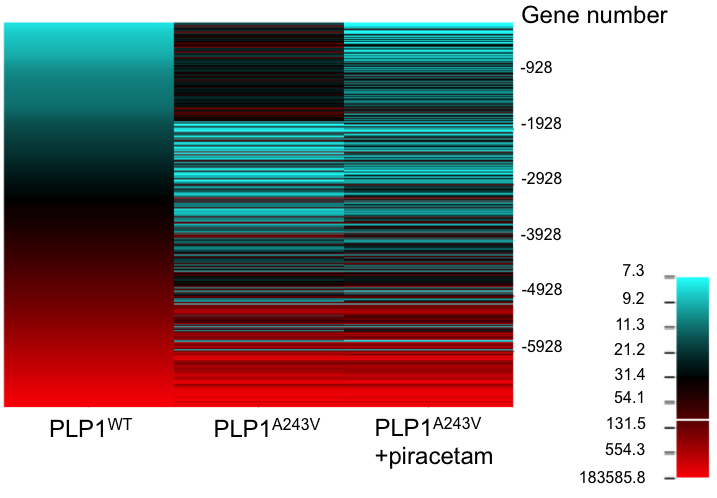


**Supplementary Figure B.** A heat map representing the color-coded expression levels of the genes in MO3.13 cells that were stably expressed in PLP1^WT^, PLP1^A243V^ and PLP1^A243V^ with piracetam. We adapted 6,927 genes for the heat map for which the expression either increased more than 2 times or decreased by less than half due to the change of a PLP1^WT^ mutation to a PLP1^A243V^ mutation. The genes in PLP1^WT^ are arranged in ascending order according to the intensity of the gene expression. The figure on the lower right shows the intensity of gene expression. The expression of each gene was converted to the heat map obtained from the CIMminer website (https://discover.nci.nih.gov/cimminer/home.do).
